# Postoperative glucocorticoid enhances recovery after endovascular aortic repair for chronic type B aortic dissection: a single-center experience

**DOI:** 10.1186/s12872-016-0234-2

**Published:** 2016-03-25

**Authors:** Mengtao Wu, Lei Zhang, Junmin Bao, Zhiqing Zhao, Qingsheng Lu, Rui Feng, Chao Song, Jian Zhou, Zaiping Jing

**Affiliations:** Department of Vascular Surgery, Changhai Hospital, Second Military Medical University, 168 Changhai Road, Shanghai, 200433 China; Department of Vascular Surgery, General Hospital of Jinan Military Command of Chinese PLA, Jinan, China

**Keywords:** Aortic dissection, Endovascular repair, Prognosis, Glucocorticoid

## Abstract

**Background:**

Thoracic endovascular aortic repair (TEVAR) has been chosen as a less invasive alternative for type B aortic dissections (TBADs). However, the therapeutic effect of TEVAR has been challenged by postoperative adverse events, which were induced by inflammatory response. Glucocorticoids have been widely used because of the powerful and effective anti-inflammatory properties. Nevertheless, the prognostic effect of glucocorticoids after TBAD patients underwent TEVAR remains unclear. The objective of this study was to assess the potential effect of postoperative glucocorticoids on the prognosis of TEVAR for TBADs.

**Methods:**

A total of 92 chronic TBADs patients underwent TEVAR with epidural anesthesia between June 2012 and June 2014 was retrospectively reviewed. The patients were stratified into dexamethasone (DXM) and non-dexamethasone group (N-DXM). The indications for TEVAR were as following: malperfusion (*n* = 28); contained or impending rupture (*n* = 17); persistent intractable chest/back pain (*n* = 32); refractory hypertension (*n* = 15).

**Results:**

No 30-day mortality and incision infection occurred in each group. The postoperative pain score on the second day was significantly higher in N-DXM group (3.60 ± 0.21 versus 4.83 ± 0.32, *P* = 0.001). The differences of white blood cell, body temperature and heart rate were pronounced in both groups judged by the peak values (13.01 ± 0.58 × 10^9^/L versus 10.04 ± 0.61 × 10^9^/L, 37.67 ± 0.08 °C versus 37.92 ± 0.09 °C and 89.06 ± 1.21 bpm versus 95.95 ± 1.70 bpm, *P* = 0.002, 0.04 and 0.001, respectively). The white blood cells in DXM group significantly increased on the second and third postoperative day (*P* = 0.009 and 0.023), while the body temperature and heart rate showed an apparent decline on the second (*P* = 0.001 and 0.028), third (*P* = 0.007 and 0.005) and fourth postoperative days (*P* = 0.024 and 0.018). However, the changes of false lumen volumes and the endoleak incidence at 3-month follow-up were comparable in the two groups. No significant difference of post-implantation syndrome was observed either.

**Conclusions:**

Although postoperative prophylactic glucocorticoids administration was unable to influence mortality, incision infection or the change of false lumen volumes, it enabled to enhance the recovery of vital signs and alleviate the postoperative pain. A prospective, randomized controlled trial has been registered (NCT02523300), which will be warranted before prophylactic administration of glucocorticoids after TEVAR procedure could be recommended in the clinical work.

**Electronic supplementary material:**

The online version of this article (doi:10.1186/s12872-016-0234-2) contains supplementary material, which is available to authorized users.

## Background

Owing to the lower risks of mortality and morbidity compared with traditional open surgery, thoracic endovascular aortic repair (TEVAR) has been chosen as a less invasive alternative for the treatment of type B aortic dissections (TBADs) in recent years [1–3]. However, the therapeutic effect of TEVAR has partly been challenged by the postoperative adverse events, which might result in disability or death [4]. The postoperative inflammatory response between stent graft and vascular wall, usually contributing to the post-implantation syndrome [5, 6], might be responsible for these adverse events [7]. But there is no consensus on how to prevent or treat the post-implantation syndrome after chronic TBAD patients underwent TEVAR until now.

Glucocorticoids have been widely used in the clinical practice by reason of the powerful and effective anti-inflammatory properties [8, 9]. Previous studies had demonstrated perioperative administration of glucocorticoids had a favorable effect on the prognosis in liver resection, abdominal surgery, hip and knee surgery, etc. [10–12]. It is worth noted that long-term and high-dose using glucocorticoids would induce adverse effects [13]. Thus, the present study sought to evaluate the clinical outcomes of short-term and low-dose prophylactic administration of glucocorticoids in chronic TBADs patients after TEVAR and elucidate the potential mechanisms involved in.

## Methods

### Study population

The study protocol complied with the declaration of Helsinki, and was approved by the Ethics Committee of Shanghai Changhai hospital. After written informed consent forms for operations were provided, a total of 306 consecutive TBADs patients underwent TEVAR from June 2012 to June 2014. The diagnosis of TBADs was confirmed by computed tomography angiography (CTA) on a 64-slice CT scanner (Siemens, Munich, Germany) in all patients. The inclusion criteria of our study were as following: 1) chronic TBADs; 2) intervention with epidural anesthesia. The exclusion criteria were acute TBADs, intervention with general anesthesia, using anti-inflammatory drugs, any trauma before TEVAR within 2 months, history of endoprosthesis implantation, history of any autoimmune disease, any type of malignancy, conservative treatment, and imcomplete data of temperature, heart rate, or white blood cell.

It should be noted that no guideline about the usage of glucocorticoids after TBAD patients underwent TEVAR was available at present. Previous studies proposed the administration of steroids could be used to reduce host biological responses in the care of post-TEVAR patients [14, 15]. On the other hand, Ker et al. demonstrated that longer fever duration was statistically associated with longer stent grafts implanted [16]. Therefore, the short-term and low-dose glucocorticoids were regularly given to prevent longer fever duration and alleviate host immune response for the patients with more than 200 mm stent grafts coverage in our center.

After reviewing the medical records, the patients were stratified into two groups: 1) the dexamethasone group (DXM), patients were prophylactically given dexamethasone (5 mg/day, intravenously) on the operation day for 3 days. Then indomethacin enteric-coated tablets (p.o., 50 mg/day) were prescribed at the end of the fasting state; 2) the non-dexamethasone group (N-DXM), patients were only given indomethacin (p.o., 50 mg/day) at the end of the fasting state. The second generation of cephalosporin was prophylactically given in all patients before operation within 30 min, and continuous blood pressure surveillance was conducted to maintain systolic blood pressure 100–120 mmHg after operation [17].

### Protocol for TEVAR procedure

All the procedures were performed in the digital subtraction angiography suite. A standard percutaneous puncture of the access artery was performed, and heparin was given intra-arterially (80 U/kg). Angiography was routinely used to identify the true lumen and primary entry tear, followed by selective catheterization of the target vessel. A stiff wire was then placed, entering into the true lumen, following which the stent grafts were advanced and deployed consecutively to cover the primary entry tear. Four stent graft systems were used in the procedures: Zenith TX2 (COOK Medical, Bloomington, IN); TAG (W. L. Gore & Associates, Flagstaff, AZ); Valiant (Medtronic, Minneapolis, MN) and Hercules-T (MicroPort, Shanghai, China) (Additional file 1: Table S1). Cerebrospinal fluid drainage was used when long-segment aortic coverage was planned. A vascular closure device was used to manage the access site after intervention. No post-deployment ballooning was used.

### Data collections

The demographics and clinical characteristics including body temperature, heart rate, white blood cell, hemoglobin, platelet, fibrinogen, activated partial thromboplastin time and prothrombin time were retrospectively collected. The date on the preoperative day and the initial 5 days after intervention were extracted and analyzed. The peak and valley values were defined as the highest and lowest values during the period of data collection.

The intensity of postoperative acute chest/back pain was assessed by another physician blinded to the study using the visual analog scale [18] at every morning until discharged from hospital. Pain scores on the second day were used to analysis.

CTA examinations were postoperatively arranged in all patients at 1- and 3-month follow-up point. Parameters were obtained with the help of dedicated three-dimensional workstation (Aquarius WS 3.7.0.13, TeraRecon Inc, San Mateo, Calif). Briefly, enhanced aortic lumen was reconstructed by volume rendering technique. Then the aortic segment where thrombosis of the false lumen exists was extracted by cropping area of interest. Volume measurements were automatically done using the “volume measure” function. The new-onset thrombosis volume in false lumen was defined as the reduced volume of aorta between the preoperative and postoperative aortic volume at the same segment.

### Definitions of post-implantation syndrome and the postoperative adverse events

Patient was diagnosed with post-implantation syndrome when the signs and laboratory tests met at least two items of the diagnostic criteria of the systemic inflammatory response syndrome. Judged by the peak values of white blood cell, body temperature and heart rate within the initial 5 days after intervention, patients were evaluated with a score of 2 or 3 depending on the number of systemic inflammatory response syndrome criteria presented.

The postoperative adverse events included cardiac events (myocardial infarction or arrhythmia), cerebrovascular events (cerebral infarction or hemorrhage), incision infection and 30-day mortality.

### Statistical analysis

All analyses were performed using IBM SPSS 20.0 (IBM Corp., Armonk, NY, USA). The values were expressed as numbers, percentages, means ± standard error or interquartile range. Categorical variables were compared using chi-squared or Fisher’s exact test, and a two-group t-test or the nonparametric Mann-Whitney U test was used to compare the continuous variables. The probability values were two-tailed and the null hypothesis was rejected for values of *P* <0.05.

## Results

### Patients’ characteristics

**Fig. 1:**
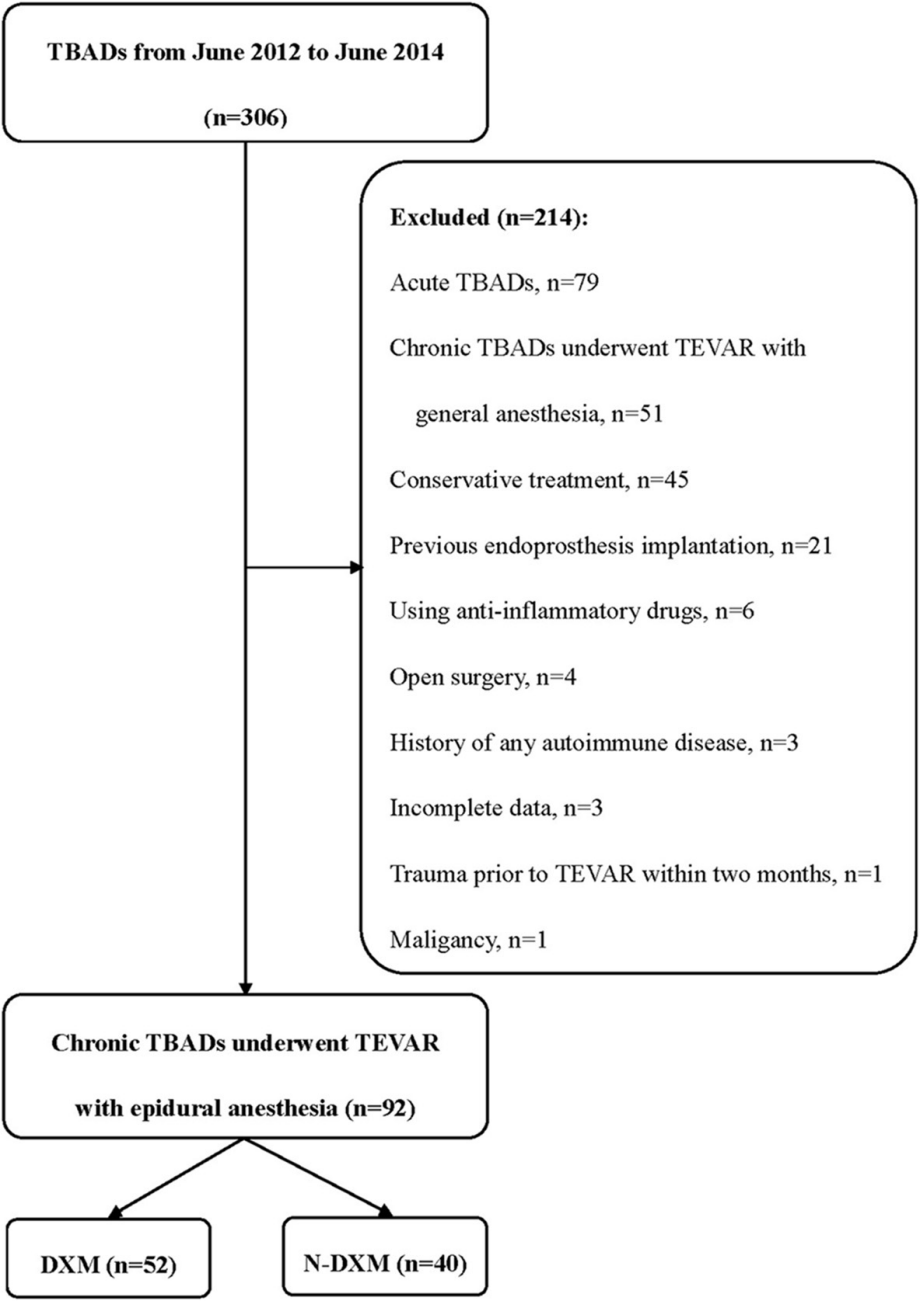
Flow diagram of included patients. *DXM* dexamethasone group, *N-DXM* non-dexamethasone group, *TBAD* stanford type B aortic dissection, *TEVAR* thoracic endovascular aortic repair

Between June 2012 and June 2014, a total of 306 consecutive TBADs patients were enrolled in our center. Of 92 were chronic dissections and intervened TEVAR with epidural anesthesia, divided into DXM (*n* = 52) and N-DXM (*n* = 40) groups (Fig. 1). The indications for TEVAR included one of the following clinical or anatomical characteristics: malperfusion (*n* = 28, 30.4 %); contained or impending rupture (*n* = 17, 18.5 %); persistent intractable chest/back pain (*n* = 32, 34.8 %); refractory hypertension (*n* = 15, 16.3 %).

Patients’ demographics were presented in Table 1. Technical success was achieved in all patients. There were no significant differences between DXM and N-DXM groups.

**Table 1:** Demographics of all patients

Variables	DXM (*n* = 52)	N-DXM (*n* = 40)	*P*
Demographic characteristics			
Age of onset, years	58.48 ± 1.85	58.74 ± 1.82	0.921
Male, *n* (%)	42 (80.8)	33 (82.5)	0.832
Body mass index, Kg/m^2^	24.17 ± 0.59	24.43 ± 0.81	0.806
Mean arterial pressure, mmHg	105.76 ± 1.99	102.76 ± 3.11	0.397
Medical history			
Smoking, *n* (%)	20 (38.5)	8 (20.0)	0.056
Cerebral disease, *n* (%)	2 (3.8)	4 (10.0)	0.236
Coronary artery disease, *n* (%)	4 (7.7)	2 (5.0)	0.604
Type II diabetes, *n* (%)	1 (1.9)	4 (10.0)	0.090
Hypertension class, *n* (%)			0.981
1	4 (7.7)	4 (10.0)	
2	9 (17.3)	7 (17.5)	
3	29 (55.8)	22 (55.0)	
ASA class, n (%)			0.968
I	2 (3.8)	1 (2.5)	
II	32 (61.6)	26 (65.0)	
III	17 (32.7)	12 (30.0)	
IV	1 (1.9)	1 (2.5)	

*DXM* dexamethasone group, *N-DXM* non-dexamethasone group

### Postoperative outcomes

No 30-day mortality and incision infection occurred in each group. There was no difference of postoperative hospital stay between DXM and N-DXM groups (6.21 ± 0.44 days versus 6.48 ± 0.36 days, *P* = 0.658). The proportions of postoperative adverse events were 1.9 and 7.5 % in DXM and N-DXM groups (*P* = 0.313), respectively. The postoperative pain score on the second day was significantly higher in N-DXM group (3.60 ± 0.21 versus 4.83 ± 0.32, *P* = 0.001). The incidence of endoleak was comparable in the two groups (1.9 % versus 5.0 %, *P* = 0.718) at 3-month follow-up point (Table 2).

**Table 2:** Postoperative outcomes

Outcomes	DXM (*n* = 52)	N-DXM (*n* = 40)	*P*
Pain score on the second day	3.60 ± 0.21	4.83 ± 0.32	0.001
Postoperative hospital stay, days	6.21 ± 0.44	6.48 ± 0.36	0.658
Postoperative adverse events, *n* (%)			0.313
Adverse cardiac events	0	3 (7.5)	
Adverse cerebrovascular events	1 (1.9)	0	
3-month follow-up, *n* (%)			0.718
Type I endoleak	0	1 (2.5)	
Type II endoleak	1 (1.9)	1 (2.5)	

*DXM* dexamethasone group, *N-DXM* non-dexamethasone group

### Systemic inflammatory response syndrome evaluation

According to the criteria of systemic inflammatory response syndrome, no significant difference of post-implantation syndrome was observed in the two groups (38.5 % versus 32.5 %, *P* = 0.555). The differences of white blood cell, body temperature and heart rate were pronounced in DXM and N-DXM groups judged by the peak values (13.01 ± 0.58 × 10^9^/L versus 10.04 ± 0.61 × 10^9^/L, 37.67 ± 0.08 °C versus 37.92 ± 0.09 °C and 89.06 ± 1.21 bpm versus 95.95 ± 1.70 bpm, *P* = 0.002, 0.04 and 0.001, respectively) (Table 3).

**Table 3:** Systemic inflammatory response syndrome evaluation and the peak value of variables during the initial 5 postoperative days

Variables	DXM (*n* = 52)	N-DXM (*n* = 40)	*P*
SIRS criteria, *n* (%)	20 (38.5)	13 (32.5)	0.555
2	17 (32.7)	9 (22.5)	
3	3 (5.8)	4 (10.0)	
White blood cell, ×10^9^/L	13.01 ± 0.58	10.04 ± 0.61	0.002
Body temperature, °C	37.67 ± 0.08	37.92 ± 0.09	0.040
Heart rate, bpm	89.06 ± 1.21	95.95 ± 1.70	0.001

*Bpm* beats per minute, *DXM* dexamethasone group, *N-DXM* non-dexamethasone group, *SIRS* systemic inflammatory response syndrome

**Fig. 2:**
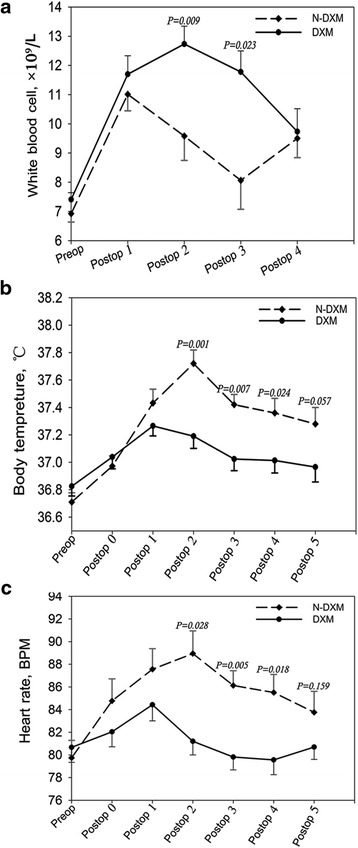
Curve charts of white blood cell, body temperature and heart rate on the preoperative day and the initial five postoperative days. *BPM* beat per minute, *DXM* dexamethasone group, *N-DXM* non-dexamethasone group, *Postop* postoperative, *Preop* preoperative

The white blood cells in DXM group significantly increased on the second and the third postoperative day (*P* = 0.009 and 0.023 respectively) (Fig. 2a). The body temperature and heart rate showed an apparent decline on the second (*P* = 0.001 and 0.028, respectively), third (*P* = 0.007 and 0.005, respectively) and fourth postoperative days (*P* = 0.024 and 0.018, respectively) in DXM group (Fig. 2b and c).

### The change of false lumen volumes

**Fig. 3:**
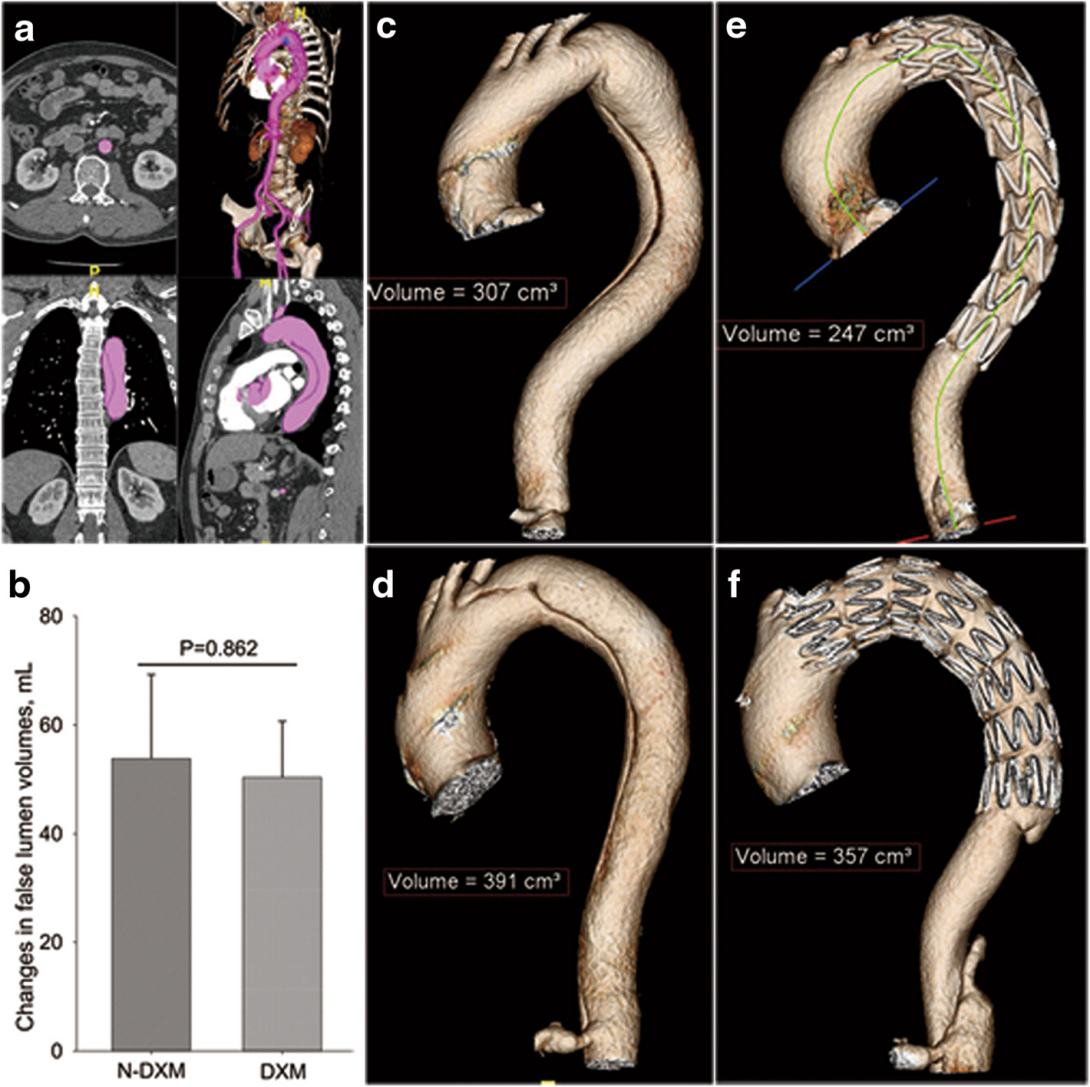
Luminal volume calculations by the dedicated three-dimensional Aquarius Workstation CT-image processing software. **a** the process of three-dimensional image reconstruction; **b** changes in false lumen volumes in N-DXM and DXM groups; **c** and **d**, preoperative aortic morphology in DXM and N-DXM groups, respectively; **e** and **f** postoperative aortic morphology in DXM and N-DXM groups, respectively. *DXM* dexamethasone group, *N-DXM* non-dexamethasone group

No difference of change of false lumen volumes was found between DXM and N-DXM groups (*P* = 0.862) (Fig. 3b). There were no significant differences in the fibrinogen, activated partial thromboplastin time, prothrombin time, platelet and hemoglobin between the two groups at the baseline and peak/valley levels (Table 4).

**Table 4:** Variables difference for new-onset thrombosis in false lumen

Variables	DXM (*n* = 52)	N-DXM (*n* = 40)	*P*
Fibrinogen, g/L			
Baseline	4.86 ± 0.26	4.29 ± 0.28	0.138
Peak value	4.44 ± 0.16	4.10 ± 0.20	0.186
APTT, second			
Baseline	35.02 ± 0.56	33.88 ± 0.77	0.225
Peak value	41.03 ± 2.52	42.36 ± 3.91	0.766
Prothrombin time, second			
Baseline	13.66 ± 0.22	13.30 ± 0.12	0.193
Peak value	13.83 ± 0.17	13.78 ± 0.16	0.834
Platelet, ×10^9^/L			
Baseline	194.81 ± 10.67	219.65 ± 13.35	0.145
Valley value	181.31 ± 10.97	178.00 ± 10.19	0.838
Hemoglobin, g/L			
Baseline	126.40 ± 2.65	131.15 ± 2.14	0.186
Valley value	115.12 ± 2.49	115.47 ± 3.02	0.931

*APTT* activated partial thromboplastin time, *DXM* dexamethasone group, *N-DXM* non-dexamethasone group

## Discussion

Many studies have concluded that perioperative glucocorticoids administration has a favorable effect on the prognosis in different lesions [10–12]. To our knowledge, the potential influence of perioperative glucocorticoids administration on the prognosis of aortic dissection after TEVAR has not been reported. In the present study, short-term prophylactic administration of low-dose dexamethasone (5 mg/day for 3 days) promoted the recovery of vital signs and alleviated the postoperative pain. However, the efficacy couldn’t be able to influence the 30-day mortality, incision infection, coagulation system, and the process of thrombosis in false lumen after chronic TBADs patients underwent TEVAR.

Body temperature and heart rate are two important vital signs, which could provide significant information about physiological condition and predict adverse events [19]. And patients with abnormal vital signs such as persistent fever and tachycardia are always associated with mortality [19, 20]. Moreover, pain is considered as the fifth vital sign [21, 22]. Acute postoperative pain might have detrimental effects on the recovery of patients, or even lead to serious morbidity or death [23]. We found that prophylactic administration of dexamethasone could improve the vital signs and alleviate the acute postoperative chest/back pain in the present study.

Although perioperative long-term and high-dose using dexamethasone might increase the risk of surgical incision infection, induce the gastric ulceration and suppress the activity of adrenal gland [13, 24], there was no enough evidence to suggest that the adverse effects was associated with short-term and low-dose administration of glucocorticoids [10–12, 25, 26], which was demonstrated in our results. The transient hyperglycemic response might have no relation with postoperative complications [27, 28]. Nevertheless, the controversies still exist about the type, time, dose, duration and strategy (prophylactic or aggressive) of glucocorticoids using [7, 14, 29, 30].

The criteria of systemic inflammatory response syndrome have been widely accepted as the diagnostic methods for post-implantation syndrome after endovascular aortic repair [7, 31, 32]. However, the proportions of post-implantation syndrome according to the systemic inflammatory response syndrome criteria were comparable in DXM and N-DXM groups. We also found administration of dexamethasone could increase leukocytosis, and lower the body temperature and heart rate to the normal level, which was regard as a “separation” phenomenon. Therefore, it might be unreasonable to evaluate the severity of post-implantation syndrome based on the systemic inflammatory response syndrome criteria. Previous study had demonstrated that dexamethasone could stimulate the neutrophils released from bone marrow and inhibit its apoptosis, which would provide an explanation for the phenomenon that the leukocyte was significantly higher on the second and third postoperative days in DXM group [33].

Glucocorticoids might increase the activity of coagulation factors in vivo [34, 35]. Perioperative usage of glucocorticoids could not only increase the activity of factor VII, VIII and XI, but also enlarge the ratio of plasminogen activator inhibitor-1 and tissue-type plasminogen activator [36, 37]. The influence of glucocorticoids on the thrombosed process of false lumen was still obscure. However, the effects mentioned above were not observed in the present study, which might attribute to the intraoperative application of heparin and persistent blood flow into the false lumen from the re-entry tear of dissection [38].

### Limitations

There are a few limitations in our study. This is a retrospective single-center study and sample size is relatively small in each group. Another important limitation was the lack of observation of the influence on inflammatory response following TEVAR due to the timing, duration and different doses of glucocorticoids administration. Thus, we could not further explain the mechanism of change in clinical status in our study. Based on the above understanding, a prospective, open, randomized, placebo-controlled trial has been registered (NCT02523300), which will be helpful to overcome the limitations, and be warranted before prophylactic administration of glucocorticoids after TEVAR procedure could be recommended in the clinical work.

## Conclusions

Although postoperative short-term prophylactic administration of low-dose glucocorticoids do not influences mortality, incision infection or the change in false lumen volumes after TEVAR procedure for chronic TBADs, it significantly promotes the recovery of vital signs and alleviates the postoperative pain. It’s worth considered to redefine the post-implantation syndrome with standardized diagnostic criteria, probably introducing determinations of some inflammatory biomarkers.

## Additional file

Additional file 1: Table S1. Detailed information of TEVAR. (DOC 35 kb)

## Abbreviations

CTA: computed tomography angiography
DXM: dexamethasone group
N-DXM: non-dexamethasone group
TBAD: type B aortic dissection
TEVAR: thoracic endovascular aortic repair
