# Supplementary material for: Postoperative glucocorticoid enhances recovery after endovascular aortic repair for chronic type B aortic dissection: a single-center experience
Source: BMC Cardiovasc Disord. 2016 Mar 25;16:59. doi: 10.1186/s12872-016-0234-2 (PMC4807598; doi:10.1186/s12872-016-0234-2)
Supplement: Additional file 1: Table S1. — Detailed information of TEVAR. (DOC 35 kb) [file 12872_2016_234_MOESM1_ESM.doc]

Additional file 1: Table S1 Detailed information of TEVAR

| Variables | DXM (n=52) | N-DXM (n=40) | *P* |
| --- | --- | --- | --- |
| TEVAR procedure |  |  |  |
| Blood loss, ml | 50 (50-100) | 70 (50-100) | 0.872 |
| Operation time, min | 94.63±5.68 | 96.25±9.42 | 0.876 |
| Type of stent-graft, n (%) |  |  | 0.207 |
| Zenith TX2 | 24 (46.2) | 12 (30.0) |  |
| TAG | 5 (9.6) | 2 (5.0) |  |
| VALIANT | 8 (15.4) | 12 (30.0) |  |
| Hercules | 15 (28.8) | 14 (35.0) |  |

DXM, dexamethasone group; N-DXM, non-dexamethasone group; TEVAR, thoracic endovascular aortic repair.
